# Differences in Polysomnography Parameters of Women in the Post and Transitional Phases of Menopause

**DOI:** 10.7759/cureus.20570

**Published:** 2021-12-21

**Authors:** Esra Dugral, Gokhan Ordu

**Affiliations:** 1 Chest Disease/Physiology, Dokuz Eylul University, Izmir, TUR; 2 Obstetrics and Gynecology, Giresun University, Faculty of Medicine, Giresun, TUR

**Keywords:** post menopausal period, menopausal transition period, hypoxia in sleep, total sleep time, obstructive sleep apnea syndrome

## Abstract

Objectives

To investigate the relationship between the changes in the main components of sleep during the menopausal transition and the postmenopausal period.

Methods

A total of 162 patients were included in the study, including 62 patients in the menopausal transition period and 100 patients in the postmenopausal period. The Epworth sleepiness scale (ESS) was applied to the patients before polysomnography (PSG). In PSG recordings, the total sleep time, sleep efficiency, rapid eye movement (REM) sleep (%), non-REM (NREM) sleep (%), apnea-hypopnea index (AHI), REM-AHI, NREM-AHI, minimum and mean oxygen saturation, oxygen desaturation time, and oxygen desaturation index (ODI) were recorded.

Results

Total sleep time (256.4±89.9 vs. 302.8±73.8, p<.03) and sleep efficiency (63.8±16.3 vs 75.6±16.0, p<.005) were significantly decreased in the postmenopausal patient group compared to the menopausal transition group. ODI, desaturation time, and desaturation percentages were significantly higher and minimum oxygen saturation was significantly lower in the postmenopausal group than in the transitional group. While mild obstructive sleep apnea syndrome (OSAS) rates in the menopausal transition group were significantly higher than in the menopausal group, moderate and severe OSAS rates were significantly higher in the menopausal group.

Conclusion

Changes in PSG measurements during the menopausal transition and postmenopausal period showed a significant effect of hormonal changes on sleep in women.

## Introduction

Sleep is an evolutionarily conserved behavioral process in many organisms and mammals, characterized by a decreased response in the cerebral cortex and muscle tone to external stimuli. Since the neuronal activity recorded by electroencephalography from the brain in wakefulness and that recorded during sleep are different from each other, sleep formation is thought to be an essential physiological process for the organism to live healthily. Although sleep time, sleep pattern, and duration vary between species, both brain and body cells are subjected to a restorative process during sleep, ensuring that the organism is in a healthy physiological and immunological functioning state [1]. For this reason, disturbances in the quality and duration of sleep may lead to the emergence of cardiovascular and metabolic diseases, as well as neurological and neurodegenerative diseases [2].

Both the menopausal transition and postmenopausal periods are periods in which sleep and sleep-disordered breathing problems are most common in women's lives. Determining how sleep and sleep-disordered breathing problems change in these two periods is of great importance for the treatment of these patients. For this reason, the definition of the intertwined menopausal transition and postmenopausal periods is very important in terms of understanding the formation of sleep problems. From a reproductive biology perspective, a woman's late reproductive period is divided into three as reproductive, menopausal transition, and postmenopausal periods [3]. Menopause is a process characterized by the last menstrual period between the ages of 40 and 59, which is a normal result of physiological aging due to the depletion of follicles in the female ovaries. While the average age of menopause is 51, the menopausal transition period begins at the age of 47 and results in menopause in an average of five years [4]. In order for us to call it menopause, the woman must not have menstruation for at least 12 consecutive months. During the menopausal transition period, women experience menstrual cycles, albeit at rare intervals. In this process, changes in the hypothalamic-pituitary and ovarian axis lead to a gradual change in serum follicle-stimulating hormone (FSH) and estradiol values ​​rather than linearly. For this reason, serum FSH and estradiol values ​​vary greatly. Therefore, there is no standard FSH and estradiol value specific to the transition period. While serum FSH levels gradually rise above ≥25 mIU/mL, a gradual decrease occurs in estradiol levels, and the patient enters menopause with complete cessation of menses within an average of two to eight years. The loss of the ability of the ovaries to produce estrogen directly in the postmenopausal period is the main reason for low estradiol levels. Small amounts of estrogen detected in the circulation occur due to peripheral conversion of androgens. The ovaries contribute indirectly to estrogen synthesis by maintaining a small amount of androgen production during menopause [5].

In healthy people, sleep shows a cyclical pattern of approximately 90 minutes, starting from NREM and progressing to rapid eye movement (REM). non-REM (NREM) sleep is divided into sub-phases as N1, N2, and N3 [6]. The N3 phase is also called slow-wave sleep (SWS) and is the deepest phase of sleep and cell regeneration takes place. A limited number of polysomnography (PSG) sleep studies have shown that woman’s reproductive periods have significant effects on sleep quality and sleep disorders. Although women in the menopausal transition period have a better-quality sleep pattern than postmenopausal women, sleep disorders and insomnia are two times more common in the late reproductive period [7-8]. Although women in menopausal transition have more total sleep time, less waking time, and better sleep efficiency in PSG analyses, it has been the subject of many studies why sleep disorders are more common in aging women [9]. Although women in the menopausal transition period are more advantageous than postmenopausal women in terms of total sleep time and SWS duration, which is deep and restorative sleep, they suffer more from sleep problems such as falling asleep, frequent night awakenings, and length of nighttime waking periods [10]. Especially, although SWS periods show little change with age, the main reason underlying women's sleep problems may be life-long changes in sex steroid synthesis and release. When the literature is reviewed, although approximately half of the patients in the menopausal transition period subjectively suffer from sleep problems, there are no data showing the deterioration in sleep quality objectively [10-12]. This study was, therefore, designed to determine traditional measures of sleep quality such as total sleep time, sleep efficiency, REM (%) and NREM (%), apnea-hypopnea index (AHI), REM-AHI, and NREM-AHI values, minimum and mean O2 saturation, desaturation time and rates as well as oxygen desaturation indices (ODI) in women with the menopausal transition period. In addition, the possible relationship between serum FSH and estradiol values and PSG measures were also tested. By choosing women with menopause as the control group, we had the opportunity to compare the PSG parameters in the menopausal transition and postmenopausal period.

## Materials and methods

Study design

Female patients who applied to the GDH Chest Diseases sleep polyclinic of the Ministry of Health between 2010 and 2013 and underwent polysomnography (PSG) were included in the study. The clinical histories of the patients were scanned retrospectively from their files, and permission was obtained from the local ethics committee for file scanning. When the sample size was calculated with the GPower 3.1 (http://www.gpower.hhu.de/) program, the total mean of the two groups was compared based on the Mann-Whitney test with the effect size of 0.50%, power of 80%, and maximum acceptable type 1 error of 5%. That calculation indicated a sample size of 154 women with a 95% confidence interval. Therefore, the medical records of 200 female patients who applied to sleep disorders polyclinic were retrospectively reviewed. Of the 200 women who underwent the medical records screening, 162 met the inclusion criteria and were enrolled in the study.

Inclusion and exclusion criteria

The diagnosis of menopause was made according to the patient's anamnesis and serum hormone levels. In patients with a uterus, having their last menstrual period one year ago was accepted as the basic menopause diagnosis criterion. In doubtful diagnostic cases, it was stipulated that FSH levels should be higher than 40 mIU/mL and estradiol levels should be less than 20 pg/mL. Patients who entered surgical menopause were considered to be in menopause from the date of surgery. In case of suspicion of having their ovaries removed, serum FSH and estradiol levels were evaluated. The inclusion criteria of the patients who participated in the study with the diagnosis of menopause were more than one year from the date of their last menstrual period. Since there is no standard age and hormone value to define the menopausal transition period, anamnesis and hormonal values should be evaluated together in the selection of patients in this group. The presence of the following was accepted as the menopausal transition period: (i) the patient's menstruation even at long intervals, (ii) serum FSH and estradiol levels varying from measurement to measurement, (iii) FSH above 25 mIU/mL but below 40 mIU/mL. Patients who met these criteria were accepted as the menopausal transition group. While 62 of the 162 participants were in the menopausal transition period, 100 of them were in the postmenopausal period. Files of patients taking hormone replacement therapy or sleeping pills and patients with uncontrolled diabetes, hypertension, severe coronary artery disease, cancer history, or severe liver or kidney failure were excluded from the evaluation. Women with oral antidiabetic therapy, insulin therapy, or diet-regulated diabetes were included in the study. Similarly, patients with drug-regulated hypertension were also included in the study. We also gathered information about chronic obstructive pulmonary disease (COPD) and tobacco consumption. Cases with COPD requiring continuous oxygen therapy were excluded from the study.

Polysomnographic parameters

The polysomnography (PSG) indications of the participants were determined as snoring, excessive daytime sleepiness, and witnessed apnea during sleep. The Epworth sleepiness scale (ESS) was used for each patient before PSG, both to evaluate daytime sleep quality and to have preliminary information about the presence of obstructive sleep apnea syndrome (OSAS). ESS is a short and easy test consisting of eight questions answered by the patient. The total score obtained as a result of the test is between 0 and 24, values below 10 are normal, while values above 10 are considered increased daytime sleepiness. The higher the increase in values, the more serious the presence of pathological sleepiness. All patients underwent overnight PSG testing, and patients with less than three hours of sleep were excluded from the study. Simultaneously with PSG, electroencephalogram (EEG) for detection of brain waves, electromyogram for muscle activities (EMG; submental EMG and tibialis EMG), electrooculogram (EOG) for eye movements, electrocardiogram (ECG) for heart rhythm, chest and abdominal belt for respiratory effort, nasal cannula and thermistor for apnea and hypopnea, pulse-oximetry for oxygen level, and tracheal microphone for snoring were used. The criteria published by the American Academy of Sleep Medicine (AASM) in 2007 were used to score respiratory events detected in sleep records. In PSG recordings obtained during the night, total sleep time, sleep efficiency, REM sleep (%), NREM sleep (%), AHI, REM-AHI, NREM-AHI, minimum oxygen saturation, mean oxygen saturation, oxygen desaturation time, and oxygen desaturation index (ODI) were recorded. The ODI is the number of oxygen desaturations per hour seen during sleep. Oxygen desaturation time is the definition, in minutes, of time spent under 90% of oxygen saturation during sleep. Apnea was defined as cessation of breathing for ≥10 seconds, and hypopnea as a decrease in breathing for ≥10 seconds with concomitant hypoxia. During apnea, airflow from the mouth or nose decreases by nearly 90%. During hypopnea, airflow is accompanied by an approximate 30% reduction in airflow and a 4% reduction in oxygen saturation, or a 50% reduction in airflow and a 3% reduction in oxygen saturation. AHI is the number of apneas+hypopneas/per hour of sleep. AHI is used to group OSAS cases according to their severity. If AHI is less than 5, OSAS is considered absent, while OSAS is diagnosed when AHI is greater than 5. AHI is grouped as mild sleep apnea if it is 5-15, moderate sleep apnea if it is between 16-30, and severe sleep apnea if it is >30 [13].

Statistical analysis

All data analysis was applied using the Statistical Package for Social Sciences software 21.0 for Windows package software (SPSS, Inc., Chicago, IL, USA). All parameters studied in the menopausal transition or postmenopausal group showed normal distributions, which were confirmed by the one-sample Kolmogorov-Smirnov test. Comparisons between the two groups were made using an independent samples t-test or Mann-Whitney U test. The relationship between the PSG measures, FSH, estradiol, and other parameters was evaluated by Spearman's correlation analysis. Data are presented as the means ± SD (Standard Deviations). A p-value of <.05 was considered statistically significant.

Ethical permission

Permission was obtained from the ethics committee of the Republic of Turkey, Ministry of Health, Buca Seyfi Demirsoy Training and Research Hospital, Non-Interventional Clinical Research with the number 2021/8-58. The study was planned according to the ethics guidelines of the Declaration of Helsinki.

## Results

The total number of patients whose files were scanned was 200, but the total number of patients included in the study was 162. Thirty-eight patients were excluded from the study due to reasons such as uncontrolled diabetes or hypertension, drug use that may affect the sleep/wake status, impaired liver or kidney function tests, COPD patients who need continuous oxygen support, and patients with a history of previous cancer surgery. While 11 (17.7%) patients in the menopausal transition group had insulin-regulated diabetes mellitus, 14 patients (14%) in the postmenopausal group were receiving insulin therapy. While 16 patients (25.8%) in the menopausal transition group had a history of hypertension regulated by the use of antihypertensive drugs, the number of hypertensive patients using drugs was 27 (27%) in the postmenopausal group. While 13 patients (20.9%) in the menopausal transition group used to smoke, 19 patients (19%) in the postmenopausal group had a history of smoking. While the number of patients with treatment-stable COPD was four (6.4%) in the menopausal transition group, it was 24 (24%) in the postmenopausal group. While the number of cases with excessive daytime sleepiness was 12 (19.3%) in the menopausal transition group, this number was 62 in the postmenopausal group (62%). Excessive daytime sleepiness and the frequency of patients with COPD were significantly higher in the postmenopausal group (p<.01, p<.03 respectively).

Demographic and PSG data of both groups of participants are shown in Table 1. The mean age of the postmenopausal patients was significantly higher than that of the menopausal transition group (55.3±7.2 vs 46.6±6.3, p<0.00). The body mass indexes (BMIs) of the menopausal transition group and postmenopausal patients were found to be similar. The mean duration of menopause of the postmenopausal patient was found to be 4.12±0.22 years. While 28 of 100 cases were surgical menopause, the remaining 72 cases were natural menopause. The serum FSH levels of the menopausal group were significantly higher than the menopausal transition group (63.3±0.11 mIU/mL vs 27.2±3.01 mIU/mL, p<0.02). Serum estradiol levels were found to be significantly higher in the menopausal transition group than in the menopausal group (26.6±2.92 pg/mL vs 10.9±6.01 pg/mL, p<0.03).

**Table 1:** Demographic and PSG parameters of each group of participants BMI: Body mass index, FSH: Follicle-stimulating hormone, AHI: Apnea-hypopnea index, ODI: Oxygen desaturation index, REM: Rapid eye movement, NREM: Non-REM, PSG: polysomnography Quantitative variables are shown as mean ± standard deviation.

Parameters (Mean± Standard Deviation)	Menopausal transition (n=62)	Post-Menopause (n=100)	p value
Age (year)	46.6±6.3	55.3±7.2	0.000
BMI (kg/m^2^)	34.6±7.0	36.2±8.6	0.282
Mean duration of menopause(year)	NA	4.12±0.22	NA
Surgical menopause	NA	28 (28%)	NA
FSH (mIU/mL)	27.2±3.01	63.3±0.11	0.020
Estradiol (pg/mL)	26.6±2.92	10.9±6.01	0.030
Epworth sleepiness scale	9.34±6.7	14.3±5.8	0.002
Excessive daytime sleepiness, n(%)	12 (19.3%)	62 (62%)	0.001
AHI	19.4±4.6	37.2±2.74	0.001
Total sleep time (min)	302.8±73.8	256.4±89.9	0.030
Sleep efficiency (%)	75.6±16.0	63.8±16.3	0.005
REM (%)	26.5±8.9	11.4±7.9	0.040
NREM (%)	88.5±7.9	89.8±9.2	0.474
NREM1	2.45±2.28	2.49±2.68	0.931
NREM2	72.6±16.0	76.1±15.4	0.272
NREM3	13.4±11.9	11.2±11.2	0.341
REM-AHI	21.6±1.8	34.6±9.2	0.007
NREM-AHI	28.1±5.0	33.5±8.9	0.067
REM-AHI	21.6±1.8	34.6±9.2	0.007
NREM-AHI	28.1±5.0	33.5±8.9	0.067
Minimum O_2_ saturation (%)	81.0±10.2	70.2±14.4	0.040
Mean O_2_ saturation (%)	92.4±5.5	90.7±5.9	0.156
Desaturation (min)	46.2±4.2	65.8±3.5	0.020
Desaturation (%)	14.4±6.9	23.9±7.4	0.003
ODI (%)	28.7±1.7	43.2±5.6	0.040

ESS results performed before PSG were significantly higher in the postmenopausal group than in the menopausal transition group (14.3±5.8 vs. 9.34±6.7, p<.002). Excessive daytime sleepiness was detected in 62 patients in the postmenopausal group and in 12 patients in the menopausal transition group, and the difference was statistically significant (p<.001). Total sleep duration (256.4±89.9 vs. 302.8±73.8, p<.03) and sleep efficiency (63.8±16.3 vs 75.6±16.0, p<.005) were significantly decreased in the postmenopausal patient group compared to the menopausal transition group. REM sleep duration was also significantly lower in the menopausal group than in the menopausal transition group (11.4±7.9 vs 26.5±8.9, p<.04). There was no significant difference between the two groups in terms of NREM sleep duration (p<.474). REM-AHI values were found to be significantly higher in the menopausal group than in the menopausal transition group (34.6±9.2 vs 21.6±1.8, p<.007). The ODI, desaturation time, and desaturation percentages were significantly higher in the postmenopausal group than in the menopausal transition group. Minimum oxygen saturation was significantly lower in the postmenopausal group than in the transitional group (70.2±14.4 vs 81.0±10.2, p<.04).

While the number of AHI>5 patients in the menopausal transition group was 15 (24.1%), it was found to be 45 (45%) in the postmenopausal patient group (Table 2). The number of patients with AHI>5 was significantly higher in the menopausal group than in the menopausal transition group (p<.001). AHI>5 cases were diagnosed with OSAS. Mild OSAS rates in the menopausal transition group (60%; n=9) were significantly higher than in the menopausal group (33.3%; n=15) (p<.001). Moderate OSAS rates were significantly higher in the menopausal group (40%; n=18 vs 26.6%; n=4, p<.002). Similarly, severe OSAS rates in the menopausal group were twice as high as in the menopausal transition group (26.6%; n=12 vs 13.3%; n=2, p<.030).

**Table 2:** Distribution of OSAS types and incidence between the two groups OSAS: obstructive sleep apnea syndrome

Parameters	Menopausal transition (n=62)	Post-menopause (n=100)	p-value
Total OSAS (AHI>5), n(%)	15 (24.1%)	45 (45%)	0.001
Mild OSAS (AHI=5-15), n(%)	9 (60%)	15 (33.3)	0.001
Moderate OSAS (AHI=16-30), n(%)	4 (26.6%)	18 (40%)	0.002
Severe OSAS (AHI>30), n(%)	2 (13.3%)	12 (26.6%)	0.030

## Discussion

Although there are many studies examining sleep disorders in postmenopausal patients, the number of studies comparing the menopausal transition period with the postmenopausal period in terms of sleep disorders is very few. Difficulties in determining the limits of the menopausal transition period clinically and laboratory have been suggested as the reason for this low number of studies. Our study is one of the most comprehensive studies that polysomnographically analyzes sleep disorders of patients in the menopausal transition period. When selecting the participants in the menopausal transition group, we used both clinical and laboratory data together to ensure that the group was homogeneous and that menopausal patients were excluded from the group. When selecting patients in the menopausal transition group, patients with FSH values ​​greater than 25 mIU/mL and less than 40 mIU/mL were included in the study. Care was taken that the serum estradiol levels of the patients should not be below 20 pg/mL simultaneously with the increase in FSH levels. Since the criteria used to determine the menopause group were clearer and more standard no difficulties were encountered. As a result, we compared the menopausal transition period, in which ovarian steroids fluctuate, and the postmenopausal period when the sex steroid values ​​decrease to the lowest level in female life.

ESS values ​​(9.34) performed before PSG were found to be significantly lower in the menopausal transition group than in the menopausal group (14.3). Since values ​​below 10 were considered normal in ESS, the values ​​we found in the transition group were within normal limits. While the number of patients in whom we found excessive daytime sleepiness in the menopausal transition group in connection with ESS was 12, we found this number was 62 in the postmenopausal group. In summary, both ESS values ​​and excessive daytime sleepiness rates of our patients in the menopausal transition group were significantly lower than in the postmenopausal group. Consistent with our results, ESS or Pittsburgh sleep quality index values ​​used to evaluate subjective sleep quality were reported to be higher in the postmenopausal patient group [14]. Possible reasons for the low number of patients with excessive daytime sleepiness in the menopausal transition group may be patient age and differences in serum estradiol and FSH values. The age of our patients in the menopausal transition group (46.6) was found to be significantly lower than in the postmenopausal group (55.3). While more estradiol is circulating in the transition group patients, the lower FSH values ​​may explain the lower rates of excessive daytime sleepiness compared to the menopausal group. It is a known fact that both normal sleep patterns and sleep problems in women are significantly affected by aging and fluctuation in ovarian steroid synthesis. The increase in the incidence of many different types of sleep disorders, especially insomnia, during the menopausal transition and postmenopausal period is the main evidence that estrogen and progesterone have an effect on normal sleep patterns [15-16]. During the transition period, the gradual decline in estrogen and progesterone production is accompanied by increases in FSH secretion that cause vasomotor symptoms. Improvement in sleep patterns following estrogen replacement therapy is a finding that supports the role of steroids in normal sleep patterns [7]. However, since mood changes, such as anxiety, depressive symptoms, and affective disorders, are common in the transition period, it is not clear whether the main problem is changes in steroid levels or the psychological state of the individual that affects sleep patterns [17-18]. The Study of Women's Health Across the Nation (SWAN) reported that increased FSH values increase the frequency of awakening from sleep during the night in menopausal transition patients [11]. The same study emphasized that decreased estrogen levels may be the cause of problems falling asleep and staying asleep.

When the parameters measured in PSG were compared between the two groups, we found that the total sleep duration, sleep efficiency, and amount of REM phase sleep were significantly higher in the menopausal transition group. When the literature data was reviewed, it was reported that total sleep duration and sleep efficiency in postmenopausal patients were significantly reduced compared to the menopausal transition group [7,16]. In the menopausal transition group, nighttime awakenings, difficulty falling asleep, and staying asleep problems are more common [11,19]. Despite the existence of subjective sleep problems in the menopausal transition period, we see that these problems are not objectively detected in PSG. With menopause, PSG parameters make it possible to determine the values ​​at which sleep and sleep breathing problems become objective. The difference in the incidence and diversity of sleep problems between the two periods and the reason why subjective complaints become objective data in PSG may be due to the increase in serum FSH levels, decrease in estradiol levels, increase in vasomotor symptoms, and mood changes with menopause. Changes in both endocrine and neuronal systems due to advancing age may also contribute to PSG parameters becoming objective data. However, in studies conducted by controlling the age factor, it has been shown that complaints of sleep and breathing problems during sleep continue [20]. Therefore, changes in PSG parameters may be related to increases in FSH levels and decreases in estradiol levels. In the SWAN study, it was reported that most of the sleep complaints in the menopausal transition period are related to FSH and estradiol changes [11]. Giving estradiol to ovariectomized rats causes a prolongation of total sleep time [21]. These data support the role of estradiol in total sleep reduction in menopausal patients. The reason for the higher REM phase sleep in the menopausal transition group compared to the postmenopausal group may be due to circulating estrogen levels. If estradiol is administered to surgically menopausal animals, it improves recovery from REM sleep in the light phase [22]. Estrogens affect sleep formation via neurons in the ventrolateral preoptic area (VLPO). Estradiol reduces the activation of sleep-stimulating neurons in the VLPO by decreasing prostaglandin D2 and mRNA expression in ovariectomized rodents [23-24]. The negative correlation between serum FSH levels and total sleep time and sleep efficiency in postmenopausal patients in our study is important evidence of the contribution of the increase in FSH levels to sleep and sleep breathing problems. The fact that the suprachiasmatic nucleus, which is the center of circadian rhythm, contains an intense estrogen receptor is also evidence pointing to the importance of estrogen fluctuations during the menopausal transition period when sleep problems are intense [25].

Apnea-hypopnea index values ​​measured in the menopausal transition group patients were found to be significantly lower than the postmenopausal group. Similarly, REM-AHI values ​​in menopausal transition patients were significantly lower than in the menopausal group. These two data show that the mechanisms that will cause both apnea and hypopnea during the menopausal transition have not been fully activated yet, and there is no problem that will prevent airflow in both the respiratory tract and respiratory brain neurons. Hypoxia does not become apparent because a normally functioning airway and the neuronal network do not allow 3% oxygen depletion in menopausal transition patients. Increasing FSH with menopause, decreasing estradiol, increasing BMI, and age-related loss of muscle strength and tone in the upper respiratory tract, and defects in the neuronal control of respiration prolong the apnea and hypopnea periods, reducing airflow and decreasing oxygen saturation while desaturation rates increase. Consistent with this, while minimum O_2_ saturation decreased in our postmenopausal patient group, desaturation time and percentage increased significantly. In addition, the number of oxygen desaturations per hour (ODI) increased significantly in the menopausal group. A total of 60 patients with AHI>5 were diagnosed with obstructive sleep apnea syndrome by evaluating the pre-PSG subjective sleep quality evaluation data and PSG analysis together. While AHI>5 in 15 patients in the menopausal transition group, the number of patients with AHI>5 in the postmenopausal group was 45. When we classified OSAS cases according to their severity, mild OSAS rates (60%) were significantly higher in the menopausal transition group than in the menopausal group (33.3%). Moderate OSAS rates were significantly higher in the postmenopausal group (40% vs. 26.6%). Similarly, severe OSAS rates in the postmenopausal group were twice as high as in the menopausal transition group (26.6% vs 13.3%). When the literature data is reviewed, it has been reported that AHI values are higher and oxyhemoglobin saturation is lower in postmenopausal patients [26].

Although the main factors in the formation of OSAS, which is characterized by snoring, upper airway obstruction, and excessive daytime sleepiness, are not known clearly, age, high BMI, and low estrogen levels are thought to be effective. However, the prevalence of OSAS continues to increase in postmenopausal women, even when confounding factors, such as BMI, age, and smoking, are excluded. Therefore, it has been suggested that the main inducer for OSAS may be decreased estradiol and increased FSH levels. It can be thought that estradiol receptors are the main culprit because of their abundance in the ventrolateral preoptic area and median preoptic nucleus as well as the suprachiasmatic nucleus and their relationship with the respiratory center and airways. The functional defect seen in the genioglossus muscle in estradiol deficiency may trigger obstructive findings in the respiratory tract [27]. Similarly, the fact that mild OSAS is higher in the menopausal transition period than in the menopausal period suggests that OSAS develops within a certain time frame. The mild OSAS development process, which starts with fluctuations in estradiol levels, completes its development in the postmenopausal period when estradiol decreases to the lowest level, and severe OSAS forms emerge. In our study, the reason why moderate and severe OSAS is more common in menopausal patients may be the net decrease in estrogen levels. The decrease in serum estradiol levels and the increase in the incidence of moderate and severe OSAS clearly support the role of estradiol in the etiology of OSAS. Although the exact mechanism is unknown, starting from the menopausal transition period, there is a significant increase in the incidence of sleep problems due to both age-related changes in BMI and muscle tone, and the cumulative effect of the decrease in ovarian sex steroid synthesis. The fact that the increase in subjective complaints about sleep during the menopausal transition period becomes objective data in PSG analyses with menopause suggests that individual characteristics and gender, as well as a decrease in sex steroids and increase in FSH levels, have important roles in the formation of sleep and the emergence of sleep disorders. This study has clinical importance in terms of comparing the PSG parameters of the menopausal transition and postmenopausal period. It is obvious that there is a need for more comprehensive studies investigating the effects of age-related and hormonal changes on sleep with a larger number of participants.

The most important limitation of this study is the use of data obtained from a single sleep center. This limits its generalizability. More accurate results will be obtained in multi-center participation. It is thought that a study that includes younger and regularly menstruating women will make a more scientific contribution.

## Conclusions

For most women, the first symptoms of menopause mark the beginning of another phase of life. This acceptance is not always calm and sometimes, it can be quite stormy. The fluctuating decrease in sex steroids in women causes not only emotional difficulties but also problems in staying healthy. In this study, clues may have been obtained that mild sleep apnea syndrome progresses to moderate and severe sleep apnea syndrome with hypoxic conditions in the postmenopausal period with the decrease in sex steroids. Increasing health problems (especially cardiovascular diseases) in the postmenopausal period may progress more rapidly with hypoxia, which is seen as a result of sleep breathing disorders. For this reason, when the first symptoms of menopause begin to appear, women's sleep disorders should also be evaluated and early diagnosis and treatment should be started before hypoxia occurs.
